# Touch Imprint Intraoperative Flow Cytometry as a Complementary Tool for Detailed Assessment of Resection Margins and Tumor Biology in Liver Surgery for Primary and Metastatic Liver Neoplasms

**DOI:** 10.3390/mps4030066

**Published:** 2021-09-15

**Authors:** Georgios S. Markopoulos, Georgios K. Glantzounis, Anna C. Goussia, Georgios D. Lianos, Anastasia Karampa, George A. Alexiou, George Vartholomatos

**Affiliations:** 1 Neurosurgical Institute, Faculty of Medicine, School of Health Sciences, University of Ioannina, 45110 Ioannina, Greece; geomarkop@gmail.com (G.S.M.); alexiougr@gmail.com (G.A.A.); 2 Haematology Laboratory-Unit of Molecular Biology, University Hospital of Ioannina, 45110 Ioannina, Greece; 3 Department of Surgery, Faculty of Medicine, School of Health Sciences, University of Ioannina, 45110 Ioannina, Greece; gglantzounis@uoi.gr (G.K.G.); georgiolianos@yahoo.gr (G.D.L.); nata_kar2007@yahoo.gr (A.K.); 4 Department of Pathology, Faculty of Medicine, School of Health Sciences, University of Ioannina, 45110 Ioannina, Greece; agoussia@uoi.gr; 5 Department of Neurosurgery, University of Ioannina, 45110 Ioannina, Greece

**Keywords:** intraoperative flow cytometry, surgical oncology, cancer diagnosis, surgical margins, hepatocellular carcinoma

## Abstract

Liver resection is the main treatment for primary and metastatic liver tumors in order to achieve long-term survival with good quality of life. The ultimate goal of surgical oncology is to achieve complete tumor removal with adequate clear surgical margins. Flow cytometry is a powerful analytical technique with applications such as phenotypic analysis and quantification of DNA content. Intraoperative flow cytometry (iFC) is the application of flow cytometry for DNA content/ploidy and cell cycle distribution analysis during surgery for tumor cell analysis and margin evaluation. It has been used for cell analysis of intracranial tumors and recently of head and neck carcinomas and breast carcinomas, as well as for tumor margin evaluation. Herein, we present a novel touch imprint iFC protocol for the detailed assessment of tumor margins during excision of malignant hepatic lesions. The protocol aims to offer information on surgical margins after removal of malignant liver tumors based on DNA content of cancer cells and to corroborate the results of iFC with that of histopathological analysis. Based on the established role of iFC in other types of malignancies, our specialized protocol has the potential, through characterization of cells in liver transection surface post hepatectomy, to offer significant information on the type of resection and tumor biology. This information can be used to effectively guide intra- and postoperative patient management.

## 1. Introduction

Carcinogenesis is a step-by-step process through which normal cells acquire genetic and epigenetic alterations and are transformed into malignant cells that subsequently create a tumor mass. Cancer is among the leading causes of human mortality worldwide, with an estimate of 19.3 million new cancer cases and almost 10.0 million cancer deaths that occurred in 2020. Future estimations are dismal since a rise of >70% in incidence/mortality has been projected for the next 20 years, with cancer-related mortality to reach >28 million new cases in 2040 [1]. The liver is the site of the development of primary and metastatic liver neoplasms for which, if left untreated, the survival is dismal (a few months). Hepatocellular carcinoma (HCC), the most common primary liver tumor, is the fifth most common cancer and the second most common cause of mortality worldwide for men. Its incidence is increasing over the years. HCC is characterized by a high mortality rate of at least 90%, since 830,000 deaths have been estimated to occur in 2020, ranking among the deadliest types of neoplasms. The main treatment that offers long-term survival with the potential for cure is liver resection and liver transplantation [2,3]. The ultimate aim of surgical oncology is to achieve complete removal of the tumor (R0 resection), along with a functional organ remnant. Surgical resection of primary liver tumors and metastatic lesions, mainly from colorectal cancer, prolongs significantly the survival, along with the good quality of life [4,5].

**Figure 1:**
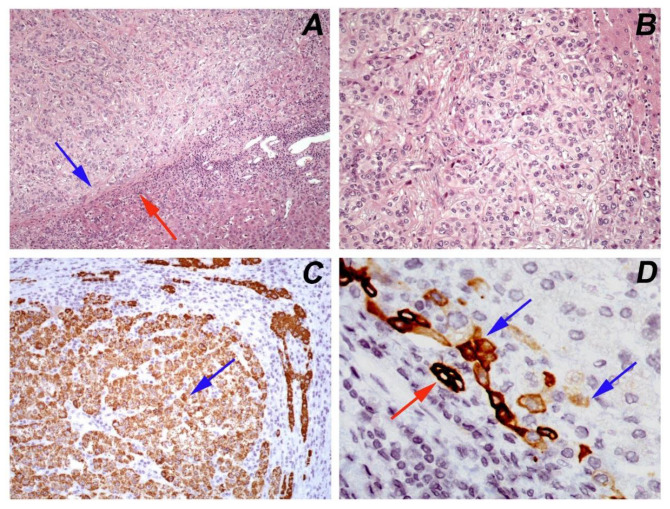
Hepatocellular carcinoma, moderately differentiated (Grade 2, WHO 2019): (**A**) a circumscribed tumor with a pseudocapsule (blue arrow) that separates it from the adjacent normal parenchyma (red arrow) (hematoxylin/eosin, ×100); (**B**) the tumor has a trabecular growth pattern and the neoplastic cells have abundant pale or eosinophilic nuclei with moderate nuclear atypia and prominent nucleoli (hematoxylin/eosin, ×200); (**C**) immunohistochemical positive expression of HepPar1 (blue arrow) confirms the hepatocytic differentiation of tumor cells (×200); (**D**) immunostaining of keratin 19 in few (<5%) tumor cells (blue arrow). The normal adjacent bile ducts serve as an internal positive control (red arrow) (×600).

Since hepatectomy remains the first line of treatment, the status of resection margins is of important significance for assessing the risk of recurrence and has a high prognostic value [6]. Histopathologic examination is the diagnostic method to evaluate microscopically the surgical margins as well as to guide the extent of resection. This information can be obtained outside the operating room by frozen tissue section examination, for a period of approximately 10–15 min per tissue sample. Moreover, detailed histopathologic analysis of HCC specimens is crucial for definite diagnosis and patient prognostication. Tumor characteristics such as size and focality, histologic subtype, histologic grade, vascular invasion, pathologic staging, and keratin 19 immunohistochemical expression are important features that predict patient prognosis, recurrence, and metastatic potential (Figure 1) [7,8].

Flow cytometry (FC) is a powerful analytical technique with several applications in phenotypic analysis and the quantification of cellular processes, such as cell proliferation and cell death [9]. The quantification of state/phenotype of a cell population is among the main advantages of FC to other methods, such as microscopy. DNA analysis is among the first widely used applications of FC, even before the development of methodologies including monoclonal antibodies [10]. Τhe concept of intraoperative flow cytometry (iFC), was based on flow cytometric quantification of DNA content/ploidy and cell cycle distribution during surgery for cancer cell analysis and margin evaluation. The rationale of iFC offered the ability for intraoperative diagnosis, which serves as an alternative to pathology evaluation of tissue sections obtained during surgery of central nervous tumors (CNSTs) [11]. A modified rapid protocol for cell cycle analysis developed in the University Hospital of Ioannina (“Ioannina Protocol”) allowed the intraoperative characterization of intracranial lesions and their surgical margins in 6 min per sample. A significant difference in G0/G1 phase, as well as in S-phase and G2/M fractions between high-grade and low-grade tumors was demonstrated. In glioblastoma patients, significant differences were observed between tumor mass and margins regarding the G0/G1 phase, the S-phase, and (G2/M) tumor fraction (tumor index), offering the potential of delineating tumor margins in gliomas [12]. As regards prognosis, recent data suggest that calculation of malignancy index based on iFC may also act as a novel prognostic factor following radiotherapy and chemotherapy with temozolomide [13]. The utility of iFC is currently being expanded beyond CNSTs into the analysis of tumor margins in several additional cancer types [14], with candidates such as head-and-neck malignancies [15,16] and breast cancer [17]. The rationale of the current study is the presentation of a novel modified protocol that uses a touch imprint on a nylon membrane of the hepatic transaction area, in order to obtain cells for flow cytometric analysis. The modified protocol is used for cancer cell characterization and margin detection during the excision of primary and metastatic liver neoplasms. The study is based on the established role of iFC by DNA-content and phenotypic analysis in CNSTs, breast-, and head-and-neck lesion-conserving surgeries, previously investigated by our research team [11,17,18,19].

## 2. Experimental Design

### 2.1. Materials

- Wipak Pouch Sterilization Steriking 11.75 In X 17.75 (Serfinity Medical Cat.No. SSU SS7);
- A total of 10 cc syringes for FNAs;
- Sterile single-pack CellTrics^®^ filters (Sysmex, Cat.no. 04-004-2323);
- BD Pharmingen™ DNA stain buffer: 1X PBS, 2% FBS, 0.1% NaN3 (pH 7.1–7.4) (BD Biosciences, Cat.no. 554656);
- BD Pharmingen™ PI/RNase A staining buffer: 125 mM propidium iodine in DNA stain buffer (BD Biosciences, Cat.no.550825);
- Ficoll-Paque PREMIUM separation (GE Healthcare, Little Chalfont, Buckinghamshire, UK Cat.no. 95038-170);
- DNA QC particles (BD Biosciences Cat.no.349523).

### 2.2. Equipment

- BD FACSCaliBur Flow Cytometer (BD Biosciences)

**Figure 2:**
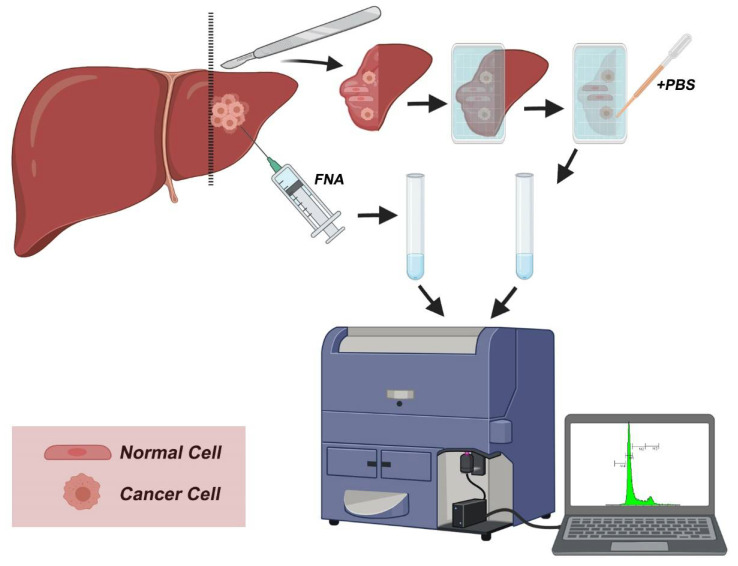
Workflow overview. During surgery, the excised surface is imprinted into a nylon membrane and cells are transferred into the imprint. Following PBS wash and filtration, the sample is stained with propidium iodide and analyzed with flow cytometry to quantify DNA content. A positive control sample is prepared from fine-needle aspirate (FNA) and analyzed in parallel to imprinted samples.

An overview of the protocol is outlined in Figure 2. Immediately after tumor excision, tumor samples were used for the creation of touch imprints into a nylon membrane. The obtained cells were rinsed in phosphate-buffered saline into a cell suspension that was further passed through a sterile filter. Cells were counted and were immediately stained with propidium iodide (125 mM). Following 3 min staining, the DNA content of samples was analyzed according to “Ioannina Protocol,” a procedure that was originally used for the analysis of DNA content in CNSTs [12]. Fine-needle aspirates (FNAs) taken from the tumor were used as positive controls to characterize the DNA content of cancer cells. In parallel, histopathologic examination of the respective tissue samples on permanent tissue sections was performed according to established diagnostic protocols. Evaluation of tumor grade was also performed according to the proposed grading systems.

## 3. Experimental Procedure

### 3.1. Preparing Cell Suspensions from Touch Imprints and Fine-Needle Aspirates for DNA Content

1. Creating cell suspension from touch imprints

Following tumor excision, touch the resected area of the liver into the membrane of a sterilized pouch (Wipak Medical) and press gently for 10 s. Rinse the cells in 0.5 mL DNA staining buffer [1X PBS, 2% FBS, 0.1% NaN3 (pH 7.1–7.4)].

2. Creating cell suspension from fine-needle aspirates (FNAs)

Obtain a 10 cc syringe used during FNA biopsy and rinse the residual material in 0.5 mL of DNA staining buffer.

From cell suspensions obtained from A or B, proceed to step 3.

3. Rake cell suspension for 10 s and pass through a sterilized filter.

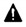
**CRITICAL STEP** The cell suspension should be minced thoroughly to obtain single cells but also gently to avoid disruption of cellular and nuclear membranes.

4. Process the cell pellet immediately by adding DNA staining solution to obtain a final Propidium iodide concentration of 125 mM. Incubate for 3 min.
5. Proceed immediately to DNA content analysis.

### 3.2. Flow Cytometric DNA Content Analysis

The following tasks should be performed before the start of the analysis:

- Evaluate instrument’s performance using DNA QC particles (BD Biosciences), using the manufacturer’s instructions.

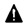
**CRITICAL STEP.** The instrument’s performance is based on the correct alignment of the cytometer. This step should not be omitted before every experiment.

- Prepare the internal control. Use ficoll-separated normal peripheral blood mononuclear cells (PBMCs). Stain 200 μL of ficoll-separated PBMCs for 3 min with PI/RNase A staining solution (125 mM of PI).

1. Use PBMC standard before every experiment, using freshly prepared ficoll-separated PBMCs, and determine their DNA content.

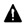
**CRITICAL STEP**. A single peak is of non-dividing cells with normal diploid DNA (G0/G1 peak) is mostly visible. The normal G0/G1 peak should be accurately defined since the geo-mean of G0/G1 peak of PBMCs is the internal control for calculating both tumor and DNA index.

2. Process DNA content analysis in a flow cytometer with 488 nm excitation and 617 nm (or compatible) emission channel. Process 5000 cells. Repeat for FNA and individual touch imprints from individual patients.

**COMMENT**: The above excitation and emission channels, compatible with PI, are among the most common in the vast majority of flow cytometers. In our case, a BD FACSCalibur (BD Biosciences) was used, with 488 nm excitation and 585/42 (FL2) emission filter was used.

**Figure 3:**
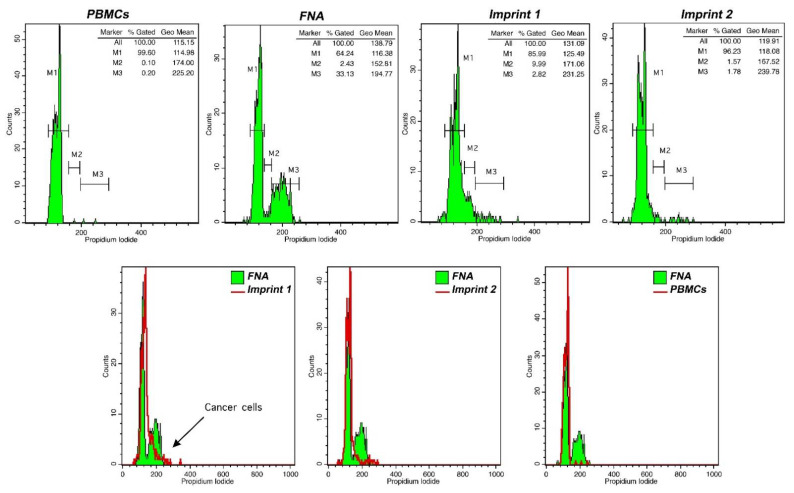
DNA analysis in touch imprint iFC. Peripheral blood mononuclear cells (PBMCs) were used as a control for DNA content evaluation. FNA samples were used to characterize malignant cells (in this case, cells with a tumor index of 35.5%). Imprint 1 represents a positive margin containing cancer cells, based on tumor index evaluation, while imprint 2 represents a negative margin. We used flow cytometry analyses in different imprints during surgery to characterize them for the presence of cancer cells. If an imprint from a patient contained cancer cells, the respective margin was indicated as positive.

3. Using flow cytometry analysis software, determine the G0/G1 geo-mean, based on PBMC fluorescence peak. Define respective areas corresponding to proliferating cells (S phase and G2/M cell cycle phases) and/or cells with altered DNA content. A typical flow cytometry analysis of FNA and touch imprint samples is presented in Figure 3.

**COMMENT 1:** A gating strategy is necessary to avoid analyzing cellular debris and/or doublets. In our analysis with CellQuest V3.1, we used an FL2 area/width gating strategy that excluded both cellular debris and doublets (Supplementary Figure S1).

**COMMENT 2:** The selection of appropriate flow cytometry analysis software may be based on the available equipment. In our example, CellQuest V3.1 was used, which is the default software for a FACSCalibul Flow Cytometer. However, there are several additional flow cytometry analysis software programs that are compatible with DNA content analysis.

### 3.3. Calculation of DNA Index and Tumor Index

1. Calculate DNA index and tumor index in FNA-obtained sample as follows:

$$DNA-index=\frac{FNA\;\left(G1\;peak\right)GeoMean}{PBMC\;\left(G1\;peak\right)GeoMean}$$

DNA index is indicative of aneuploidy. DNA index >1.05 or <0.95 is considered as aneuploid. Anything in between is not informative.

Tumor−index=SUM(S%+G2/M%)

The tumor index is informative of proliferative potential since it is the collective fraction of cells in the S and G2/M phases. A tumor index >5% indicates a proliferative cell population, indicative of cancer.


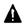
**CRITICAL STEP**: The aforementioned calculations are critical for the characterization of cancer cells in FNA samples. In the next step, the detection of cancer cells in touch imprints is based on PBMC peak analysis and the calculations made in the FNA sample.

2. Perform DNA index and tumor index calculations for individual FNA samples. The presence of cancer cells in imprint samples is based on the characterization made for the respective FNA sample.

**COMMENT 1:** Calculation of tumor index in individual imprints is made with reference to positive control/FNA. In diploid tumors (DNA index = 1) M2 and M3 from FNA and PBMCs coincide. The calculation of tumor index in aneuploid tumors (DNA index ≠ 1) is based on M2 and M3 that are drawn on FNA sample analysis.

**COMMENT 2:** In diploid tumors, pathological cell discrimination is based on tumor index in the respective imprint, wherein M2 and M3 represent proliferating cells (Figure 3).

In aneuploid tumors, the discrimination between normal and cancer cells is solely based on DNA index. In such cases, cancer cells in an imprint would appear as a distinct aneuploid peak, determined by the analysis of the respective FNA sample.

## 4. Results and Discussion

Flow cytometry analysis offers several advantages for tumor diagnosis. Firstly, it is a cell-based methodology, maximizing sensitivity down to the single-cell level. Secondly, it is fast and offers the possibility of analysis of whole-cell populations. In that way, clonal expansion of cancer may be monitored as subpopulations with distinct genetic characteristics. The intraoperative use of flow cytometry, based on the aforementioned advantages, contributes toward the precise characterization of tumor margins and the potential for complete tumor removal, which is the main goal in surgical oncology. In the field of liver surgery, the information provided from flow cytometry on the presence of cancer cells in the hepatic transection surface could change the intraoperative management with the performance of further liver resection if the future liver remnant is adequate and could identify the patients who are at high risk for local recurrence. In this way, adjuvant treatment could be offered in a few weeks postoperatively in cases who are at high risk for recurrence, and a very close follow-up should be implemented.

Touch imprints have been extensively used in the cytological evaluation of tumor cells in several types of cancer, including primary and metastatic breast cancer [20,21], melanoma [22], as well as hepatic malignancies [23]. Touch imprint iFC offers several advantages to assist clinical management. DNA content analysis based on “Ioannina Protocol” [12] can be performed within 6 min from sample collection, rapidly providing information to the surgeon, while flow cytometry analysis can accurately characterize cancer cells taken from fine-needle aspirates (FNAs). The touch imprint covers the surface of the resected liver, allowing analysis of cancer cells for the whole resected area. The availability of characterized cancer cells in the margin area may offer the possibility for further molecular analysis.

The suggested protocol of iFC is used in an ongoing study that evaluates tumor margins of hepatic resection specimens. Histopathologic analysis performed in parallel is used as the gold standard for malignant cells’ characterization.

Preliminary results are presented in Table 1, as a guide when utilizing touch imprint iFC during hepatic resection. We used flow cytometry analyses in different imprints during surgery to characterize them for the presence of cancer cells. If an imprint from a patient contained cancer cells, this was indicated as positive in Table 1. Our results contain the analysis of samples from nine patients. A pathological assessment revealed three patients with HCC grade 2 and six patients with metastatic tumors, mainly (5/6) colorectal adenocarcinomas (CRC) and one gastroesophageal adenocarcinoma (GEA). FNA analysis in HCC revealed a DNA index of 1 and tumor index of 10–35 %, confirming the existence of malignant cells. In metastatic disease, three FNA samples contained cells with DNA index ≥1.5, indicative of aneuploidy. In the other three samples, the tumor index was ≥7. TI-iFC revealed a total of three positive margins. In these margins, touch imprint iFC results corroborated microscopic evaluation, which detected tumor cells at the closest distance of ≤ 7 mm.

The presented protocol provides a roadmap on the use of touch imprint intraoperative e flow cytometry during hepatectomies. To be sure, the presented results are preliminary and have to be further elucidated in large clinical trials. Moreover, the clinical significance of our findings needs to be explored in long-term patient follow-up. A key concern in applying TIiFC is the precise imprinting of the tissue surface into nylon membrane, in order to analyze an adequate number of cells using flow cytometry. For this reason, the details of the presented protocol have to be accurately followed. Another shortcoming of the proposed study is the fact that the presence of cancer cells in positive margins would need to be independently confirmed using other sensitive techniques, such as next-generation sequencing, in order to avoid the possibility of false-positive conclusions. The molecular profiling of cancer, including individual driver mutations, has diagnostic, prognostic, and, in some cases, therapeutic value [24,25]. In some described cases in the bibliography, liver cell dysplasias exhibit aneuploid cell populations [26,27]. Since DNA index is a property used to characterize cellular malignancy with intraoperative flow cytometry, the possibility to characterize liver cell dysplasia as a malignant tumor may be a possible limitation on the use of TIiFC protocol. Analysis of larger sample size is necessary to validate this hypothesis.

The gene mutation landscape has the potential to be associated with iFC parameters, in order to verify the presence of cancer cells. The presence of cancer cells in margins that are found at a distance from a solid tumor would agree with the “seed-and-soil” hypothesis [28]. Based on this conceptual framework, first described by Stephen Paget in 1889, some cancer cells may act as “seeds” of metastasis crosstalk with their microenvironment, which acts as “soil”. The hepatic tissue is a permissive microenvironment for the invasion and migration of tumor cells of both primary and secondary neoplasia, thus acting as “fertile soil”.

The methodological advances in next-generation sequencing are currently leading to the development of precision cancer medicine [5,24]. The molecular characterization of tumors becomes the new standard and is now a critical factor for the therapeutic and clinical management of patients [25]. As regards HCC, molecular profiling has aided the classical pathology approach, providing novel insights into the mechanisms of HCC development. We believe that future integrative analysis of TI-iFC data, based on the protocol we describe, along with immunohistochemical data and the genetic mutation landscape of tumors, will enhance the diagnostic–prognostic significance of iFC to further assist patient management during and after surgery.

## Figures and Tables

**Table 1:** Results from iFC and pathologic evaluation in resected primary and metastatic liver noeplasms.

#	DNA Index	Tumor Index	TI-IFC ^1^	Pathologic Diagnosis ^2,3^	Microscopic Evaluation ^4^
**1**	1	15	+	HCC, Grade 2	U (<1 mm)
**2**	1	10	−	HCC, Grade 2	U (>10 mm)
**3**	1,5	20	+	CRC *, Grade 2	U (7 mm)
**4**	1	13	−	CRC *, Grade 2	U (5 mm)
**5**	1,6	12	+	CRC *, Grade 2	U (4 mm)
**6**	1	13	−	CRC *, Grade 2	U (>10 mm)
**7**	1,9	4	−	GEA *, Grade 2	U (4 mm)
**8**	1	7	−	CRC *, Grade 3	U (>10 mm)
**9**	1	25	−	HCC, Grade 2	U (>10 mm)

^1^ Touch imprint intraoperative flow cytometry results, + positive margin, − negative margin. ^2^ Cancer nomenclature: HCC: hepatocellular carcinoma, CRC: colorectal adenocarcinoma, GEA: gastroesophageal adenocarcinoma, * metastatic disease. ^3^ Grading Scale, Grade 1 (well-differentiated tumors), Grade 2 (moderately differentiated tumors), Grade 3 (poorly differentiated tumors). ^4^ Microscopic evaluation of surgical parenchymal margins, I: involved by invasive carcinoma, U: uninvolved by invasive carcinoma (closest distance of invasive carcinoma from the margin is indicated in mm).

## Data Availability

Not applicable.

## Supplementary Materials

The following are available online at https://www.mdpi.com/article/10.3390/mps4030066/s1. Supplemetary Figure S1: Gating strategy to exclude cellular debri and doublets. Gating is done on a dot-plot based on Propidium iodide (FL2) fluorescence pulse area (Y-axis) and width (X-axis). Gate R2 corresponds on proliferating cells, excluding debri (lower left part of dot-plot) and doublets (right part of the histogram). Two representative dot-plots from PBMCs and imprint are presented.
