# Supplementary material for: Touch Imprint Intraoperative Flow Cytometry as a Complementary Tool for Detailed Assessment of Resection Margins and Tumor Biology in Liver Surgery for Primary and Metastatic Liver Neoplasms
Source: Methods Protoc. 2021 Sep 15;4(3):66. doi: 10.3390/mps4030066 (PMC8482241; doi:10.3390/mps4030066)
Supplement: Supplementary file 1 [file mps-04-00066-s001.zip › mps-1340770-supplementary.pdf]

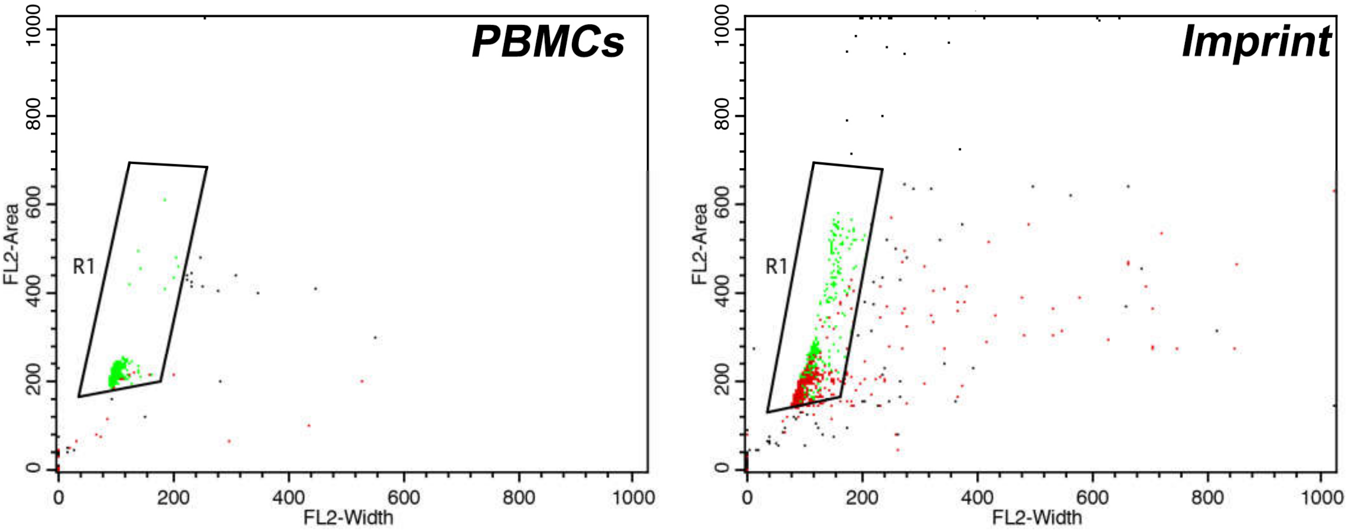

Supplemetary Figure S1: Gating strategy to exclude cellular debri and doublets. Gating is done on a dot-plot based on Propidium iodide (FL2) fluorescence pulse area (Y-axis) and width (X-axis). Gate R2 corresponds on proliferating cells, excluding debri (lower left part of dot-plot) and doublets (right part of the histogram). Two representative dot-plots from PBMCs and imprint are presented.
